# Home-based care for umbilical cords of neonates by family caregivers in Mpumalanga province, South Africa

**DOI:** 10.4102/hsag.v30i0.2676

**Published:** 2025-04-04

**Authors:** Happiness P. Sabina, Ntsieni S. Mashau, Bumani S. Manganye

**Affiliations:** 1 Department of Public Health, Faculty of Health Sciences, University of Venda, Thohoyandou, South Africa

**Keywords:** family caregivers, neonatal home-based care, neonatal care, umbilical cord care, neonates

## Abstract

**Background:**

Multiple substances have been applied to neonates’ umbilical cords and have yielded detrimental results on neonates’ health status.

**Aim:**

The study aimed to explore and describe home-based care for umbilical cords of neonates by family caregivers.

**Setting:**

The study was conducted at Waterval community, a village under Dr J.S Moroka local municipality in Nkangala district in the Mpumalanga province.

**Methods:**

A qualitative exploratory, descriptive research study design was used to explore home-based care for neonates’ umbilical cords. The target population was family caregivers who had been caring for the umbilical cords of neonates at home. Non-probability and purposive sampling were done, and individual in-depth interviews were used for data collection. The sample size of 18 participants was determined by data saturation. Thematic analysis was utilised to analyse the data.

**Results:**

The following main themes emerged during data analysis: substances applied on the umbilical cord, beliefs associated with umbilical cord care, the effectiveness of health education provided on discharge from the community health centre and the healing process.

**Conclusion:**

The findings of the study revealed that various harmful substances were applied to the umbilical cords of neonates, and these practices were influenced by cultural beliefs.

**Contribution:**

The findings of the study brought to light that indeed, the application of substances that have not been recommended by the South African guidelines on neonates’ umbilical cord does lead to delayed umbilical cord separation and healing and has the potential to cause neonatal infections.

## Introduction

In low-income countries, the incidence rates of neonatal infections related to umbilical cord range between 2 and 7 per 100 live births. These umbilical cord infections are caused by pathogens from the environment or through contact with caregivers or healthcare workers (Oyebade et al. 2023). This is further supported by Odabasi and Bulbul (2020), who, in their study, asserted that, across the globe, infections are the major cause of death in neonates because of diversified cord care practices determined by cultural customs. Most of these infections gain entry to the neonates’ bloodstream through the umbilical cord owing to the substances applied during umbilical cord care (Odabasi & Bulbul 2020).

According to the World Health Organization (WHO 2014), sub-Saharan Africa had the highest neonatal mortality rate in 2022, recording 27 deaths per 1000 live births. The deaths of neonates within the first month of life are higher in developing countries than in developed countries. As reported in the 2022–2023 District Health Barometer by Health Systems Trust (2023), South Africa recorded 12 073 neonatal deaths. Among these, 9137 deaths (75.7%) occurred during the early neonatal period, within the first 7 days of birth, while 2936 deaths (24.3%) occurred between eight and 28 days of birth. Neonatal deaths are still escalating despite the recommendations that were made by the World Health Organization in 2014, which were published to be adopted by all countries (Odabasi & Bulbul 2020). South Africa has adopted chlorhexidine cord cleansing and dry cord care during the neonatal period, as the World Health Organization recommended in 2014 (Department of Health [DoH] 2014).

In developed countries, the care and management of umbilical cords have moved towards dry umbilical cord care. Dry umbilical cord care is also used in home deliveries, community settings and resource-limited populations such as Native American communities. Prophylactic topical agents, such as povidone-iodine, are also applied to the umbilical cord (Ans et al. 2023).

There are several factors linked to early neonatal sepsis in developing countries, and these include home deliveries, non-sterile births, inappropriate umbilical cord care practices and late recognition of conditions that pose a risk of infection in the mother or baby (López-Medina et al. 2020). The sustainable development goals (SDGs) target 3.2 recognised reducing child mortality as a critical societal and health system goal. This means targeting preventable deaths in children and neonates (Massyn, Barron & Day 2020).

The guidelines currently practised in South Africa for umbilical cord care are the newborn care charts (Department of Health [DoH] 2014). Castleberry and Nolen (2018) argued that no studies have addressed the recommended interventions. There are several antiseptics used to prevent infection of the neonatal umbilical cord. Only 70% alcohol and 4% chlorhexidine have been studied extensively. However, other methods based on traditional customs, such as mixtures of herbs, ash, breast milk or oils from different sources, are less commonly used and have not been researched well (Castleberry & Nolen 2018).

Green et al. (2020) revealed that in Africa, umbilical cords are often cut with non-sterile razor blades during home births, and substances such as charcoal, baby powder, chicken excreta and oils such as cooking oil and used motor oil are applied on the cord stumps for medicinal and protective purposes in home settings. Applying these substances delays the detachment of the umbilical cord and causes umbilical cord infections (Green et al. 2020). Neonatal deaths from infections still occur, raising concerns about caregiver practices.

## Research methods and design

A qualitative research approach was used in this study, with an exploratory, descriptive and contextual study design. This study aimed to explore, describe and understand home-based care for umbilical cords of neonates by family caregivers. The study’s target population was family caregivers caring for a neonate’s umbilical cord.

### Setting

The study was conducted at Waterval community, a village under the Dr J.S. Moroka local municipality in the Nkangala district in the Mpumalanga province. Nkangala district is the smallest of the three districts in Mpumalanga province and had a population of 1 590 000 in 2023, with annual growth of 1.51% from 2019 to 2024 (Health Systems Trust 2023).

According to the Ndlovu & Padarath (2023), the Nkangala district municipality recorded 270 neonatal deaths in the 2022–2023 District Health Barometer. In comparison, Ehlanzeni had 384 neonatal deaths, and Gert Sibande reported 371. However, the exact number of neonatal deaths caused by infections remains unknown.

### Population and sample

All the participants were selected using non-probability purposive sampling. The Waterval Community Health Centre (CHC’s) delivery register was used to select study participants. The inclusion criteria were all female family caregivers caring for a neonate’s umbilical cord not more than 6 months before the commencement of the study. Participants were of any age above 18 years; they were all females and permanent residents at Waterval. Only female participants were included because mothers, grandmothers, aunts and sisters usually help with caring for neonates. Those who were ill or admitted to a hospital during the study were excluded, and family caregivers of neonates older than 6 months were excluded. Family caregivers of neonates who have spent their first week of birth in the hospital were also excluded from the study.

### Data collection method

Data were collected through individual, in-depth interviews. Participants were given an informed consent form with an information letter that provided details about the study and informed them that the participation was voluntary. Participants who could not read were provided with an oral explanation of the contents of the information letter outlining their rights during the study. Those willing to participate in the study were asked to sign the consent form. Interviews were conducted in a convenient setting for the participants, which was their home. One central question, ‘How do you care for the umbilical cord of a neonate at home?’ was asked and followed by probing questions.

The interviews were conducted in the IsiNdebele language, which the participants were comfortable with. The first author conducted interviews and was familiar with the participants’ language. The first author was trained and guided on how to conduct qualitative interviews by the supervisors. Data saturation was reached at participant number 14 when participants were repeating the information; however, interviews were continued to confirm data saturation and stopped at participant number 18 because there was no new information from the participants. Data collection for this study started in April 2023 and ended in May 2023. Field notes were used to record non-verbal actions observed by the researcher during data collection.

### Data management and analysis

Thematic analysis was used for data analysis. All the interviews were tape-recorded. First of all, the data were translated from IsiNdebele to English and back-translated from English to IsiNdebele by a language expert to ensure consistency and correctness of the information. The study used the six steps of thematic analysis process defined by Braun et al. (2023) to analyse the data. The first author listened to the tape-recorded interviews and read and re-read the transcripts. Categories of meanings that belong to the same experience were made, leading to identifying themes.

Emerging themes were clustered, and connections between themes were identified. These themes were then clustered into sub-themes. The same process was repeated for all the participants’ transcripts. The first author compared the clustered themes across the transcripts. Subordinate themes for all transcripts were clustered into superordinate themes, which were then clustered into comprehensive concepts for the sample. After all the participants’ transcripts had been considered, the first author wrote the themes with their sub-themes. An independent coder re-coded the data from the transcripts and compared them with the themes to ensure consistency.

### Measures to ensure trustworthiness

Trustworthiness was ensured by following the criteria used in qualitative research, including credibility, dependability, transferability and confirmability. The researcher ensured credibility by establishing rapport with the participants through prolonged engagement. The interviewer visited the participants to make appointments for data collection. The participants were visited so that they could ask questions about the study and the data collection process and for them to feel free in the presence of the interviewer on the day of data collection. Telephone calls were also made to establish rapport with the participants. Dependability was ensured by providing a detailed description of the research process and how decisions were made for methods used to conduct data collection and analysis to allow other researchers to assess the consistency of the findings and to replicate the study.

A thick description of the research procedures was provided for the study to be replicable and to enable them to compare the findings with what happens in real-life situations that they have encountered. This was done to ensure transferability. To ascertain that the study’s findings are the result of the participants’ experiences, the researcher included an in-depth methodological description. Confirmability was ensured through an audit trail, which involved keeping the data records, including field notes, to allow experts and other researchers to ensure that the findings are a true reflection of what transpired during data collection. An independent coder re-coded the data to ensure the themes were derived from the data.

### Ethical considerations

Ethical clearance number (FHS/22/PH/23/0303) was obtained from the University of Venda before the study was conducted. The Mpumalanga Department of Health also permitted the study to be conducted with the approved (reference no: MP_202303_010). The Waterval Community Health Centre and the Ndzundza Mabusa Traditional Council gave written permission to conduct the study in the Waterval community. All the participants were provided with an information sheet, all research procedures were explained, written informed consent was obtained, and participation was voluntary. Consent was obtained to audio record the conversations during data collection. All the collected data were kept safe in a locked cupboard, and a secret password protected the soft copies.

## Results

Four themes and sub-themes emerged from data analysis. The socio-demographic profile is shown in Table 1.

**TABLE 1:** Socio-demographic profile of participants.

Participant number	Age (years)	Relationship with baby	Previous experience with cord care
Participant 1	29	Mother	No experience
Participant 2	23	Mother	Experienced
Participant 3	35	Mother	Experienced
Participant 4	31	Mother	Experienced
Participant 5	38	Aunt	Experienced
Participant 6	42	Grandmother	Experienced
Participant 7	35	Mother	Experienced
Participant 8	38	Aunt	Experienced
Participant 9	23	Mother	No experience
Participant 10	30	Mother	Experienced
Participant 11	27	Mother	Experienced
Participant 12	41	Mother	Experienced
Participant 13	40	Mother	Experienced
Participant 14	43	Aunt	No experience
Participant 15	38	Aunt	Experienced
Participant 16	53	Grandmother	Experienced
Participant 17	46	Grandmother	Experienced
Participant 18	28	Mother	No experience

### Socio-demographic profile

Table 1 presents the socio-demographic information of the study participants. All the participants were females aged between 21 years and 53 years. The socio-demographic data collected about the participants included their age, relationship with the newborn baby and their experiences of umbilical cord care to understand the context of umbilical cord care.

### Themes and sub-themes

The themes and sub-themes were formulated based on the findings from the interviews. The following four themes emerged from the responses: substances applied to the umbilical cord, beliefs associated with umbilical cord care, the effectiveness of health education provided on discharge from the community health centre and the healing process. Themes were further divided into sub-themes, as shown in Table 2.

**TABLE 2:** Themes and sub-themes.

Main themes	Sub-themes
1. Substances that are applied to the umbilical cord.	1.1. The type of substance applied to the umbilical cord. 1.2. Effectiveness of using the substance. 1.3. Frequency of application.
2. Beliefs associated with umbilical cord care.	-
3. Effectiveness of health education provided on discharge from the community health centre.	3.1. Provision of health education on discharge. 3.2. Application of the provided health education.
4. The healing processes.	4.1. The duration of the healing process. 4.2. Complications during the healing process. 4.3. Management of the complications of the healing process.

#### Theme 1: Substances that are applied to the umbilical cord

The findings of this study revealed that participants were using different substances to apply on the umbilical cord of the neonates.

**Sub-theme 1.1. The type of substance applied to the umbilical cord:** The types of substances applied to the umbilical cord were inconsistent; some participants applied Vaseline mixed with burned paper, Grandpa powder (a powdered medication for pain relief, mostly used for headaches, toothaches, etc.) or coal:

‘We used Vaseline with a burned newspaper; we mixed the ash of the paper with the Vaseline and applied it on the umbilical cord.’ (Participant 8)

‘We collected excreta of mice and added water to them so that they can be a soft mixture, then applied them on the umbilical cord.’ (Participant 4)

Some participants explained that they used ‘incema’ (grass used to make mats, dried, burned, and its ashes mixed with water and excreta of mice), a common mixture used in KwaNdebele to apply on neonates’ umbilical cords:

‘She [*her mother*] would tell me to mix excreta of a mouse with “incema” and water mix it well then apply to the umbilical cord and cover with a cloth.’ (Participant 5)

Meanwhile, baby’s mother, said:

‘After bathing her, I would put small amounts of sugar on the sides of the umbilical cord; I would put it three times or every time I changed her nappy.’ (Participant 11)

Most participants explained how they used the different mixtures to apply on the umbilical cords of neonates:

‘I applied surgical spirit, Vaseline mixed with Grandpa on the umbilical cord, then, I put a R5 coin on the umbilical cord and tied a cloth around the stomach to press the coin.’ (Participant 12)

Another participant said:

‘After bathing the infant, I would spit saliva around the umbilical cord and cover with a cloth that I tie around the baby’s tummy.’ (Participant 16)

‘I applied haarlemansis [*oil drops that contain a high amount of alcohol, which is used for the treatment of restlessness, sleeplessness, constipation, and discomfort associated with winds*] to the umbilical cord of my baby and thereafter I tied a cloth around the tummy to press the umbilical cord.’ (Participant 6)

**Sub-theme 1.2. Effectiveness of using the substance:** Looking at the study findings, it was apparent that using most of the topical substances that kept the umbilical cord moist was not effective. This extended the healing process, and many participants returned to using surgical spirit as they had been taught:

‘I used the bandage to cover the umbilical cord because I did not want the nappy to touch the cord of the baby… but when I went to the clinic for three days visit, the nurse said I should remove the bandage and not use it anymore. so, I started believing what the nurse told me that the umbilical cord should be dry and not wet. When I was applying the bandage, it was always moist and did not look like it was healing after that it dried out and detached.’ (Participant 9)

Meanwhile, other participants argued that they did not see a difference between using the recommended methods of dry cord care and the traditional methods because the time it took for the umbilical cord stump to fall off was almost the same:

‘I did not see much difference between the first and second method, both umbilical cords took less than 7 days to fall.’ (Participant 2)

**Sub-theme 1.3. Frequency of application:** Most of the participants indicated that they were applying substances to the baby’s umbilical cord three times per day, and some participants mentioned that this was part of the health education provided by the healthcare workers at the Waterval community health centre. Some applied substances only when they changed the neonate’s diaper, while others applied the substances frequently:

‘I would use a cotton wool with the surgical spirit and apply on the umbilical cord every now and then, maybe three times a day, but I did not really count how many times I did it.’ (Participant 7)

‘What I love about this method is that it is very fast, you apply the mice faeces today, tomorrow the umbilical cord will be detached.’ (Participant 4)

Another participant said:

‘I think I applied surgical spirit three times a day, and when I changed the nappy.’ (Participant 3)

#### Theme 2: Beliefs associated with umbilical cord care

The participants’ responses showed that they had different beliefs associated with the substances that they used to apply on the umbilical cord stump of the neonates. Some participants believed that cold, air and evil spirits enter the neonate’s body through the umbilical cord stump, and these could affect the neonate later in life, and they may have stomach problems:

‘My grandmother said the baby is feeling cold, so we should cover the umbilical cord.’ (Participant 10)

Another participant said:

‘Yes, the cloth prevents spirits from entering the baby’s body through the umbilical cord, so you must tie it until three months when it has healed completely.’ (Participant 1)

‘Cover the umbilical cord with a bandage to prevent air from entering the umbilical cord. If you don’t, when the child grows up, he will have stomach cramps and will pass a lot of gas. We use this method in my country. I am originally from Zimbabwe, but I have stayed here for a long time now; that is also how my mother cared for our umbilical cords; it is a traditional thing that we practice as Zimbabweans.’ (Participant 7)

‘Instead, the baby looked in pain when we have not tied the cloth, it has like she had cramps in the stomach, and it made sounds, but the opposite happened when the stomach was tied.’ (Participant 11)

#### Theme 3: Effectiveness of health education provided on discharge from the community health centre

Most participants demonstrated that they are knowledgeable about the recommended umbilical cord care; however, some still adhere to their traditions and cultural beliefs because of family and community influence. They believe that they must keep their traditions within the generation and pass them down to the next.

**Sub-theme 3.1. Provision of health education on discharge:** Some participants indicated that they tried to follow the health education provided on discharge from the clinic. However, because of insufficient funds to buy the surgical spirit, they resorted to the traditional methods of caring for the neonate’s umbilical cord:

‘Before I was discharged from the Waterval clinic, I was told by the nurse who helped me deliver my baby that I should buy surgical spirit and cotton wool to clean the umbilical cord with. My mother and I did not have money to buy the things we were told to buy, so we thought of other ways to care for the cord.’ (Participant 1)

A participant confirmed that the nurses involved her and her daughter when they were providing health education on umbilical cord care on discharge from the clinic; she said:

‘I was there to pick them up, so the nurses involved me when they were teaching her how to care for the umbilical cord, so I listened very carefully because, with both my children, my mother helped with taking care of their umbilical cords. I didn’t know anything; she never showed me how to do it, and she is late now, so I was nervous about it …’ (Participant 17)

‘I grabbed that opportunity to learn, and when we got home, I left them and went to the chemist to buy surgical spirit and cotton. when I came back, we started immediately because they did not apply it at the clinic; they don’t have the spirit; we first bathed the baby, then applied the surgical spirit with the cotton, cleaned the pegs [*cord clamp*] as well and let it dry.’ (Participant 17)

The participants explained that the Waterval Community Health Centre nurses do provide them with health education on discharge. Some of the patients utilise the health education about applying surgical spirit on the umbilical cord at home, and some use it after they have applied harmful substances on the umbilical cord, which has caused complications.

Some participants knew the recommended cord care method; however, they preferred to follow their cultural beliefs rather than the health education they received from the clinic. They believed that the recommended method was for white people and that black people must follow their own cultural beliefs:

‘The nurses shout at us if we go there with a covered umbilical cord, so when you go to the clinic, you don’t cover it, you will do it after [*laughing*]. I am being honest, though; we know the nurses say it is wrong, but this is our culture; we cannot just take what the whites are teaching you at school; we must also follow our roots, sister.’ (Participant 7)

**Sub-theme 3.2. Application of the provided health education:** Some participants did apply the health education provided to them; they mentioned that the Waterval CHC nurses educated them, and they were told to use surgical spirit and apply it on the umbilical cord stump using cotton wool three times per day:

‘I used surgical spirit to clean the umbilical cord of my baby three times a day. Yes, the nurses showed us how to do it.’ (Participant 3)

In this study, some participants reported that they could not effectively implement the recommended cord care practices because of financial constraints that prevented them from purchasing surgical spirit, as advised by their nurse. Conversely, other participants shared that they initially used traditional methods for cord care, but when the umbilical cord failed to heal and dry properly, they opted to follow the nurses’ recommendations:

‘Oh! You don’t know! We started with a mixture of soap and butter … eh … it was not easy, the baby’s umbilical cord was not drying; we then changed to surgical spirit.’ (Participant 15)

#### Theme 4: The healing process

The duration of the healing process ranges from 1 to 2 weeks, with a mean of 3 days. Most of the participants who used surgical spirit said it took less than a week for the umbilical cord stump to fall off, while some who used other methods said it took longer than a week, and some neonates had complications during the healing process.

**Sub-theme 4.1. The duration of the healing process:** The study findings show that the healing process took less than 7 days for those who were using surgical spirit on their neonate’s umbilical cord stump to fall off and heal:

‘After using the surgical spirit to clean the umbilical cord, it took a few days to fall off.’ (Participant 3)

However, in the case of participants who used traditional methods, it took about 2 weeks for the healing process. This caused complications or infections for the neonates that led to either hospital admissions or a shift in their practice of umbilical cord care by pursuing the methods recommended by the CHC health professionals:

‘So, we started believing what the nurse told us that the umbilical cord should be dry and not wet. When I was applying the bandage, it was always moist and did not look like it was healing. Mmh, it took about seven days for the umbilical cord to fall off.’ (Participant 8)

**Sub-theme 4.2. Complications during the healing process:** Some participants said they will never use their traditional methods again because of the complications they have encountered. Most participants believed that if the umbilical cord was not moist, it caused the baby pain and complications. Those who used traditional methods of cord care indicated that it was a family tradition passed from generation to generation:

‘We covered the umbilical cord with a white cloth around the tummy to maintain moist, eh! We were taught by our grandmother, in our family, we do it to all babies.’ (Participant 6)

Some participants who used the traditional methods explained that the umbilical cords did not heal well, and some were referred to the hospital for further management:

‘I used a cloth to cover the baby’s umbilical cord, but then the umbilical cord got rotten, then I went to the clinic for a day visit; the sister said he had jaundice, then we were referred to the hospital, and they told me that I did the wrong thing.’ (Participant 10)

Some participants were afraid to take the baby to the clinic when the umbilical cord was septic after following the traditional methods of cord care instead of following the instructions provided by the healthcare providers on discharge from the clinic:

‘Hey sister, I regret using the coal and Vaseline. My child’s cord was rotten; it had a smell, and it was red around it; it was becoming a wound. I was scared to go back to the clinic because I did not follow their instructions, so the nurses would be angry and shout at me. So, I decided to ask for money from my child’s father and bought the surgical spirit and cotton wool.’ (Participant 1)

**Sub-theme 4.3. Management of the complications of the healing process:** To some participants, returning to the health facility was their solution, which is a good choice because this can prevent further complications associated with the neonate’s infections:

‘They took over; they used cotton and surgical spirit; they said what I did on the umbilical cord would make me and the baby stay for a long time in the hospital because we are taught how to care for the umbilical cord, but when we get home, we do our own thing.’ (Participant 10)

Most of the participants who used traditional methods said they would not use the substances again as they ended up using surgical spirit to reverse the complications:

‘Hey, the first one got me worried, and I had to waste money to go to the hospital, now my grandmother knows that I don’t want her method. [*Shaking her head*], I have learned that if I get another child, I will not use any bandage.’ (Participant 10)

## Discussion

The findings of this study revealed that the participants had used various substances on the umbilical cords of neonates to enhance healing. These practices are performed by covering the umbilical cord stump with a clean white cloth or bandage or compressing the umbilical cord stump with a coin. Similarly, a study conducted by Kyololo and Kipkoech (2023) in Kenya revealed the use of potentially harmful substances such as saliva, ash and dust on the cord stump.

The findings from the study conducted in Ghana corroborate the findings of the current study. The majority of mothers and caregivers used substances that were not recommended by WHO, such as shea butter, herbs, salt, ground banana peels mixed with shea butter, sand mixed with salt, chalk mixed with salt and strong antiseptics such as Dettol, while only few used the recommended cord dressing of using surgical spirit and let the cord dry by not covering it (Asiedu et al. 2019). In contrast, a study conducted in Rwanda by Uwingabire et al. (2020) revealed that the majority of mothers followed the recommendation by WHO for dry cord care by allowing the cord to air dry naturally and not applying any substance. However, in the same study, few mothers applied other substances, such as excreta from rats, ashes, saliva and Vaseline, although they also applied breast milk, which is recommended because of its anti-bacterial effect, which destroys microorganisms and minimises their multiplication.

A study conducted by Karumbi et al. (2013) in Nigeria revealed that participants used various substances to apply on the cord area such as traditional herbs combined with cooking oil or water that had been used to wash an adult woman’s private parts, ash, breast milk, fluid from pumpkin flowers, powder ground from local trees, cow dung, ghee, and saliva. Many of these substances may be detrimental to the neonates. Applying a moist substance and covering the umbilical cord seemed to cause a delay in the healing process. Therefore, these practices indicate that people still use harmful ways to care for the umbilical cord.

In the current study, the majority of participants practised potentially harmful traditional cord care although the babies were delivered at a healthcare facility. In contrast, the findings of a study conducted in Ethiopia revealed that potentially harmful substances were applied to babies who were delivered at home (Merga et al. 2022). In the same study, it was explained that those who delivered at a healthcare facility were provided with health education on infant care during the antenatal clinic and after delivery. Asiedu et al. (2019), in their study conducted in Ghana, noted that the type of dressing applied to the umbilical cord was influenced by both the place of birth and the caregivers’ recommendations. They found that nurses predominantly determined the choice of approved dressings, while unapproved options were often influenced by grandmothers, traditional birth attendants or friends of the family. A study by Dessalegn et al. (2022) in Ethiopia revealed that most mothers who delivered at home applied unrecommended substances such as butter and Vaseline compared to those who delivered at a health facility and attended the antenatal clinic.

The findings of this study indicated that the use of non-recommended substances on the umbilical cord was linked to an increased risk of umbilical cord infection. Similarly, a study conducted in Ghana by Turyasiima et al. (2020) found that applying substances such as saliva and ash to the neonate’s cord in an attempt to promote healing was significantly associated with umbilical cord infection. In the same study, it was further revealed that most of the study participants who applied potentially harmful substances on the neonate’s umbilical cord were from rural areas with less information on the recommended cord care practices compared to the participants from the urban settings. This had a great effect on the care of the umbilical cord by mothers. In contrast, a study conducted by Abbaszadeh, Hajizadeh and Jahangiri (2016) in Iran demonstrated that applying breast milk to the umbilical cord resulted in a shorter duration of cord stump separation when compared to applying topical chlorhexidine. Additionally, breast milk was found to effectively reduce the incidence of infection to a level comparable to that achieved with chlorhexidine. This effectiveness is attributed to the antimicrobial properties of breast milk, which contains antibodies that enhance the neonate’s immune system.

The findings of this study indicated that participants applied substances to the cord stump three times daily, as well as during nappy changes. A study conducted in northern Uganda by Opii, Wani and Kule (2022) found that mothers also cleaned their babies’ umbilical cords thrice daily, using clean, cool, boiled water.

There are many beliefs and norms associated with umbilical cord care, and many participants in the current study believed that the umbilical cord should be covered with a bandage to prevent air from entering the neonate’s stomach, as this will cause abdominal problems for the neonate in the future. Participants demonstrated that umbilical cord care has a symbolic meaning; hence, they believed the tradition must be passed down to the next generation.

This finding indicates that traditional methods are still followed throughout the African continent, and they are being taught to the younger generations. This further emphasises the need for health education. A study conducted in Tanzania showed that people resist behavioural changes in umbilical cord care. They demonstrated strong beliefs about the significance of applying a substance on the umbilical cord to promote healing (Shamba et al. 2013). Participants in a study conducted by Turyasiima et al. (2020) in Ghana explained that they applied potentially harmful traditional substances to facilitate cord healing and separation.

A study conducted by Arabiat et al. (2019) in Jordan found that culture strongly influences how people raise their children and guides what should be done immediately after birth. People who belong to the same culture may perform the same cultural practices, which has significant meaning to them. These norms and values are incorporated into daily activities and expectations by society.

Almost all the participants from this study confirmed receiving health education on umbilical cord care from the healthcare workers in the health facility where they delivered their babies. The main problem was that applying the provided health education conflicted with the participant’s cultural practices and beliefs. Most participants explained that cleaning the umbilical cord with surgical spirit and exposing it to dry worked well. Nonetheless, there is still a need for health education because the participants did not have knowledge about the disadvantages of not performing the recommended methods of umbilical cord care.

In a study conducted in Kenya by Mulwa, Mbugua and Karonjo (2023), most participants indicated that they received adequate information on neonatal cord care. However, some participants reported that the information they received was insufficient. In the current study, some participants started applying the knowledge they gained from health education after noticing signs of infection or complications around the umbilical cord stump. Health facilities can, for example, display posters with demonstrations of how to perform the recommended umbilical cord care procedure and pictures of complications caused by applying harmful substances displayed in waiting areas. Most participants and health workers aim for the umbilical cord stump to fall off quickly because that reduces the possibility of bacterial infections as the stump atrophies and prevents the risk of infection.

Participants acknowledged that covering the umbilical cord with a cloth or bandage prolonged the healing process by keeping the umbilical cord moist instead of dry. A moist environment is a breeding ground for bacteria, which causes infections. Most participants who used the traditional methods said it took a week to 2 weeks for the healing process, which increases the chances of bacterial infections on the umbilical cord.

The WHO (2017) recommends the application of 4% chlorhexidine to the umbilical cord stump daily during the first week of life for neonates delivered at home in areas with high neonatal mortality rates. Clean and dry cord care is advised for neonates delivered in healthcare facilities, as well as in low neonatal mortality settings at home. In these cases, chlorhexidine should only be considered an alternative to using harmful traditional substances on the cord stump. Therefore, to facilitate the healing and drying process of the umbilical cord, no substances must be applied to the cord stump to keep it moist. Linhares et al. (2019) also emphasised that the umbilical cord stump should be kept clean and dry during the healing process. The management of umbilical cord care has undergone several changes over the years because the WHO discourages covering cord stumps and recommends dry cord care in all settings (WHO 2017).

The findings of this study revealed that even within a single community, the types of substances applied to umbilical cords vary because of cultural and traditional diversity. Some participants followed the advice from the health professionals at the health centre and had satisfactory outcomes.

### Limitations of the study

The study was conducted in one community, and a more significant number of participants from different locations may yield more results. However, a full description of the methodological processes carried out in this study was provided to ensure transferability.

## Conclusion

The findings of the study revealed the need for more studies to be conducted about the mechanism of the traditional substances. However, this should be conducted in a respectful and culturally sensitive manner. The findings highlighted that health education was effective because the participants knew the advantages of the recommended methods of dry cord care, and most of them used it. However, irrespective of having received health education, some participants continued to use traditional methods and only used the recommended methods as a last option after noticing that the traditional remedies were causing complications to the umbilical cord. Immediate application of the recommended methods of umbilical cord care is essential because it is a prophylaxis against infections. The principle that says prevention is better than cure must be applied in this case.
